# High Doses of Daunorubicin during Induction Therapy of Newly Diagnosed Acute Myeloid Leukemia: A Systematic Review and Meta-Analysis of Prospective Clinical Trials

**DOI:** 10.1371/journal.pone.0125612

**Published:** 2015-05-20

**Authors:** Qiang Gong, Lixin Zhou, Shuangnian Xu, Xi Li, Yunding Zou, Jieping Chen

**Affiliations:** Department of Hematology, Southwest Hospital, Third Military Medical University, Chongqing, China; Queen's University Belfast, UNITED KINGDOM

## Abstract

The right dose of daunorubicin (DNR) for the treatment of newly diagnosed acute myeloid leukemia (AML) is uncertain. Previous trials have shown conflicting results concerning the efficacy of high or low doses of daunorubicin to induction chemotherapy for newly diagnosed AML. A systematic review and meta-analysis was conducted to resolve this controversial issue. We compared the efficacy and safety of high doses of daunorubicin (HD-DNR) and traditional low doses of daunorubicin (LD-DNR) or idarubicin (IDA) during induction therapy of newly diagnosed AML. Data of 3,824 patients from 1,796 articles in the literature were retrieved and six randomized controlled trials were analyzed. The primary outcomes were overall survival (OS), disease-free survival (DFS), and event-free survival (EFS). The secondary outcomes included complete remission (CR), relapse, and toxicity. The meta-analysis results suggest that comparing HD-DNR with LD-DNR, there were significant differences in CR (RR = 1.19, 95%CI[1.12,1.18], p<0.00001), OS(HR = 0.88, 95%CI[0.79,0.99], p = 0.002), and EFS (HR = 0.86, 95%CI [0.74, 1.00], p = 0.008), but not in DFS, relapse, and toxicity. There were no statistically significant differences in any other outcomes between HD-DNR and IDA. The analysis indicates that compared with LD-DNR, HD-DNR can significantly improve CR, OS and EFS but not DFS, and did not increase occurrence of relapse and toxicity.

## Introduction

Acute myeloid leukemia (AML) refers to a kind of clonal hematopoietic stem cell disorders which is the common type of leukemia[1]. Its overall incidence is approximately 3.7 per 100,000 persons with a median age of 67[2]. In the past years, the improvement of survival and complete remission for AML is mainly dependent on dose augmentation of standard drugs. But although after several decades of investigation, the efficacy of high and low doses of daunorubicin to induction chemotherapy for newly diagnosed AML has still showed conflicting results.

As of today, the standard induction therapy of AML remains as the “3+7” strategy, namely, three days of anthracycline (eg. daunorubicin, 45–60 mg/m², idarubicin, 12 mg/m²,or the anthracenedione mitoxantrone, 10–12 mg/m² and seven days of cytarabine at a dose of 100–200 mg/m²)[3]. This regimen achieves 60%-80% of complete remission but only 40%-45% of overall survival for younger adults, and other intervention has not been showing better[4, 5]. For patients who are aged over 60, 40%-50% present a good performance status in complete remission, but cure rates are lower than 10%, and median survival is less than 1 year[6, 7]. The condition is even worse for those who have shown a poor performance status or unfavorable cytogenetics[2].

Up to now, the appropriate dose of daunorubicin induction therapy of AML is uncertain. Some large randomized controlled trials have provided vital data on efficacy and safety of high doses of daunorubicin[8–13]. However, the right dose of daunorubicin for individual treatment is still uncertain. The highest level of clinical evidence, namely, systematic review and meta-analysis has not been done, and the precise effects of adopting high doses of daunorubicin were not known. This systematic review and meta-analysis pooled current available data from randomized controlled trials which compared the efficacy and safety of high doses of daunorubicin (HD-DNR) with low doses of daunorubicin (LD-DNR) or idarubicin (IDA), in order to provide clinical evidence and to help physicians to choose appropriate strategies for individuals in AML induction therapy.

## Methods

This work was carried out following the Cochrane Handbook of systematic reviews. We used Review Manager (version 5.2) to analyze all statistics and make the diagrams including forest plots and risk of bias assessment.The work was reported based on PRISMA (Preferred Reporting Items for Systematic Reviews and Meta-Analyses) statement[14].

### Search strategy

A computer-based search on Pubmed, Embase, and Cochrane library was performed according to a detailed search strategy from the inception to April 2014. Some other databases (Science Direct online, Chinese clinical trial register, China National Knowledge Internet, BMJ, MDconsult, greynet, wolters Kluwer, system for information on grey literature in Europe) were also screened out by using “daunorubicin”,”acute myeloid leukemia” and “randomized controlled trial” as the search items. Other relevant references, conferences and proceedings (European Hematology Association, American Society of Hematology, and the American Society of Oncology) from 1995 to 2014 were also screened. We also search international clinical trials register websites for ongoing clinical trials and unpublished clinical data (https://clinicaltrials.gov/, http://www.isrctn.com/).There is no restriction on the language of published articles.

### Inclusion and exclusion criteria

Studies with the following criteria were included: participants were previously untreated AML patients according to WHO or FAB (French-American-British) diagnostic criteria, whose liver, kidney, heart, and lung had adequate function or ECOG performance status were between 1–3, regardless of age or ethnicity. Studies with any one of the following two comparisons were included: (i) HD-DNR vs. LD-DNR, and/or (ii) HD-DNR vs. other anthracyclines. Based on NCCN recommendation, we chose a cumulative dose of 180mg/m^2^ of daunorubicin as the cut-off value for high and low doses of daunorubicin. The total dose of idarubicin was of 30–50 mg/m^2^[2]. There were no limits on study location, follow-up period, the length of induction therapy, or drug administration methods. Studies that evaluated treatment effects in patients with myelodysplastic syndrome or secondary malignant tumors which may influence the effect of chemotherapy and basic status of patients were excluded for less heterogeneity. Studies aimed not to evaluate effectiveness and safety of HD-DNR were also excluded because of lack of outcomes that we needed.

### Study selection and data collection process

Study selection was conducted by two authors independently according to the pre-defined inclusion criteria. Agreement of both reviewers determined the final inclusion of relevant studies. If consensus could not be achieved, a third author would be involved. According to standardized data extraction form, data collection was conducted by the same two authors independently. Disagreement was resolved by discussion. Main contents of data extraction form consisted of study details, study eligibility, study characteristics, risk of bias assessment and results. Primary outcomes included overall survival, disease-free survival, and event-free survival. Secondary outcomes were complete remission, induction death, relapse, and toxicity.

### Definition of outcomes and risk of bias in individual studies

Outcomes were defined by the recommendation of International Working Group [15]. complete remission was defined as the presence of the following: < 5% blasts in bone marrow; ≥ 1.0× 10^9^ /L neutrophils and ≥ 100× 10^9^ /L platelet in peripheral blood, and without evidence of extramedullary leukemia. Relapse after complete remission was defined as recurrence of leukemic blasts in the peripheral blood, or reappearance of >5% blasts in bone marrow not attributable to any other cause (eg, bone marrow regeneration after consolidation therapy), or appearance of extramedullary leukemia. Overall survival was measured from the date of random assignment until death which was due to any cause or was censored at the last follow-up. Event-free survival referred to the interval from entry into the study to the date of treatment failure, relapse from complete remission, or death due to any cause. Disease-free survival for patients who achieved complete remission was calculated from the date of complete remission until the date of relapse or death of any cause or was censored at the last follow up.

Risk of bias was assessed for each individual study by two independent reviewers according to Cochrane Handbook for Systematic Reviews of Interventions (version 5.1.0).

### Statistical analysis

To systematically evaluate the treatment, dichotomous variables were pooled by using risk ratio (RR) as an effective measurement under the fixed-effect model while survival data were pooled by using hazard ratio (HR) under the fixed-effect model, with 95% confidence intervals respectively. If HR was not reported directly, it would be extracted from relevant Kaplan-Meier curves or calculated by data transformation[16]. Heterogeneity pooled studies was calculated by using I^2^ of chi-square-based Q test and ranked as low (<30%), moderate (30–50%), or high (>50%)[17]. Heterogeneity was considered statistically significant if P<0.10 (according to Cochrane Handbook for Systematic Reviews of Interventions). Considering some significant prognosis factors, subgroup analyses for overall survival, event-free survival, disease-free survival, and complete remission were performed, based on age and cytogenetic risk classifications if relevant data were available. As the total number of included studies was 6 (<10), funnel plots were inappropriate to present. Meta-analyses were performed on the basis of intention-to-treat (ITT) principle. All statistical analyses were carried out using the Review Manager (version 5.2).

## Results

### Characteristics of patients and trials

**Fig 1:**
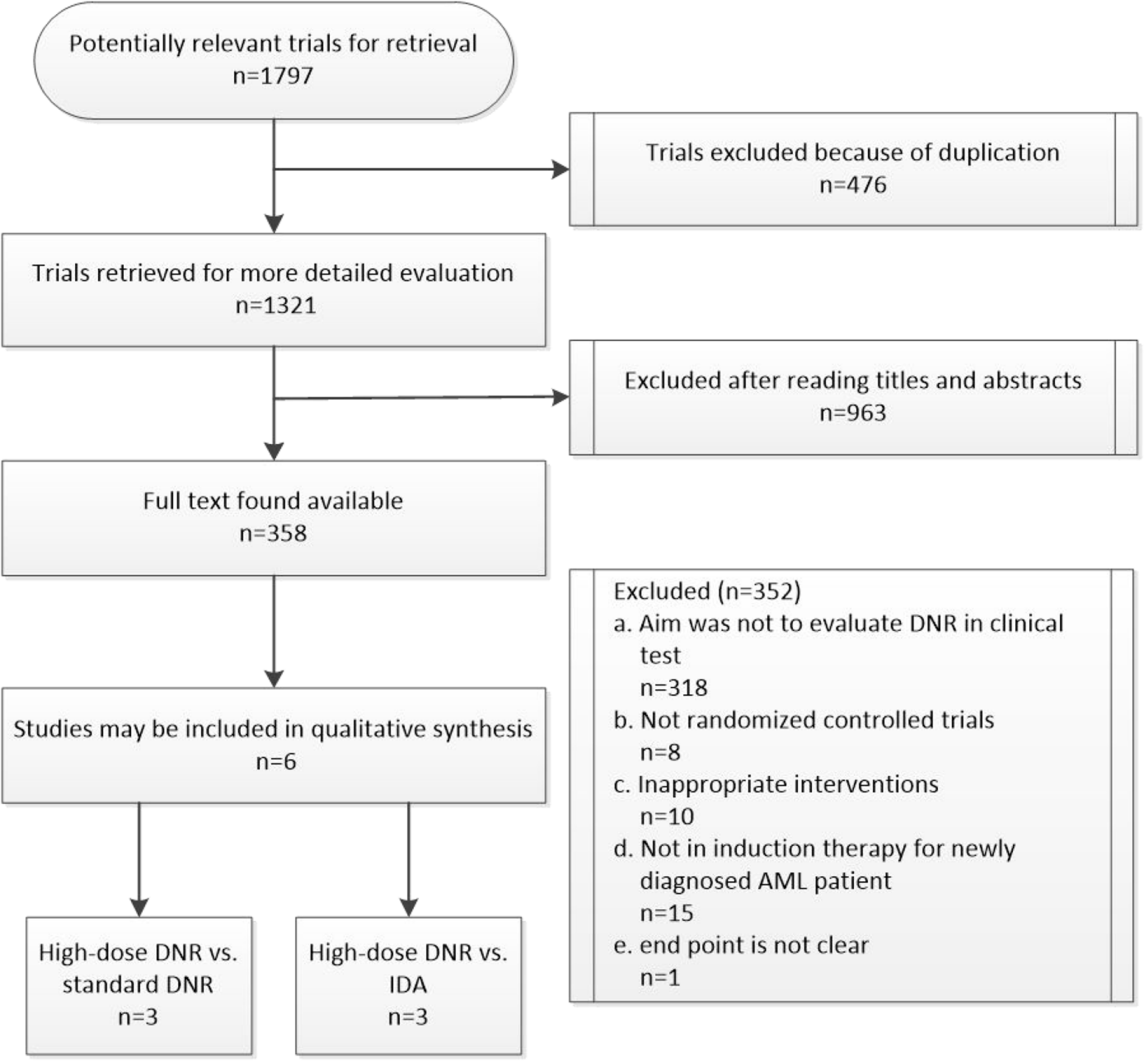
Flow diagram depicting identification and retrieval of eligible studies for inclusion.

Based on the pre-defined search strategy, 1,796 potentially relevant trials were found from the primary retrieval. After the process of advanced retrieval (Fig 1), six randomized controlled trials met the eligibility criteria in total in which 3,824 patients were treated with newly diagnosed AML and they were included in meta-analysis (Table 1).

**Table 1:** Characteristics of the six randomized, controlled trials included in meta-analysis.

Study	No. of patients (HD-DNR/LD-DNR or HD-DNR/IDA)	Range of age (median age,years)	HD-DNR/LD-DNR ratio (mg/m²×days)	HD-DNR/IDA ratio (mg/m²×days)	Other chemotherapeutic drugs combined with (mg/m²×days)	Consolidation regimen		Median follow-up (months)
						HD-DNR	LD-DNR/IDA	
**Löwenberg et al,2009**	813(402/411)	60-83(67)	90×3/45×3	-	Ara-C(200×7)	Mylotarg TM CMA-676	Mylotarg TM CMA-676	40
**Fernandez et al,2009**	657(289/293)	17-60(48)	90×3/45×3	-	Ara-C(100×7)	Mylotarg TM CMA-676	Mylotarg TM CMA-676	25.2
**Lee et al,2011**	383(194/189)	15-60(-)	90×3/45×3	-	Ara-C(200×7)	Ara-C+DNR	Ara-C+DNR	52.6
**Creutzig et al,2013**	521(257/264)	<18¶	-	80×3/12×3	Ara-C+VP-16¤	HAM©	AI/2-CDA or AI®	60
**Ohtake et al,2010**	1057(525/532)	15-64(47)	-	50×5/12×3	Ara-C(100×7)	Ara-C+MIT or hAra-C†	Ara-C+MIT or hAra-C	48
**Pautas et al,2010**	468(156/155/157)*	50-70(60)	-	80×3/12×3/12×4	Ara-C(200×7)	Ara-C+DNR	Ara-C+IDA	49

DNR, daunorubicin; IDA, idarubicin; Ara-C, cytosine arabinoside; MIT, mitoxantrone; VP-16,etoposide; Mylotarg TM CMA-676, gemtuzumabozogamicin

HD-DNR, high doses of daunorubicin; LD-DNR, low doses of daunorubicin.

©: HAM means high-doses cytarabine[3g/m²]/mitoxantrone.

®: cytarabine[0.5g/m²]/idarubicin/2-chloro-2-deoxyadenosine or cytarabine[0.5g/m²]/idarubicin.

*: HD-DNR/IDA3/IDA4.

¶: There was no detailed information about total range of age or total median age.

¤: There was no detailed information about doses of Ara-C or Etoposide.

†: hAra-C means high doses of Ara-C.

Among included studies, three compared high doses of daunorubicin with low doses of daunorubicin while the others compared high doses of daunorubicin with equivalent doses of idarubicin. The 6 trials were classified into two groups, namely Group 1 (HD-DNR vs. LD-DNR)[9–11] and Group 2 (HD-DNR vs. IDA)[8, 12, 13].

In Group 1, 885 patients were assigned to be treated with high doses of daunorubicin while 893 patients were treated with low doses of daunorubicin. In Löwenberg et al study (2009), elder patients ranging from 60 to 83 (median age, 67) were included. In Fernandez et al (2009) and Lee et al (2011) studies, younger patients aged 1760(median age, 48) and 15–60, respectively, were included. The patients were all administered with high doses of daunorubicin at 90mg/m²×3 days or low doses of daunorubicin at 45mg/m²×3 days in induction therapy. Median follow-up of these three trials was 40, 25.2, and 52.6 months, respectively [9–11].

In Group 2, 938 patients were assigned to receive high doses of daunorubicin therapy and 1,108 patients were treated with idarubicin in induction therapy. Three trials in Group 2 involved patients at three different age groups. Creutzig et al (2013) conducted a clinical trial on pediatric AML patients younger than 18 years old. Ohtake et al (2010) studied adult patients between 15 and 64 years old (median age, 47), while Pautas et al (2010) recruited older AML patients aged 50–70 (median age, 60). For drug administration in induction therapy, the assigned daunorubicin dose in high doses of daunorubicin arm was 80mg/m²×3 days in two trials[8, 12], and 50mg/m²×5 days in one trial[13]. The subjects in idarubicin arm were allocated idarubicin at 12mg/m²×3 days, except that Pautas et al (2010) set another arm with idarubicin at 12mg/m²×4 days. Median follow-up of the studies was 60, 48, 49 months respectively [8, 12, 13].

### Risk of bias within studies

**Fig 2:**
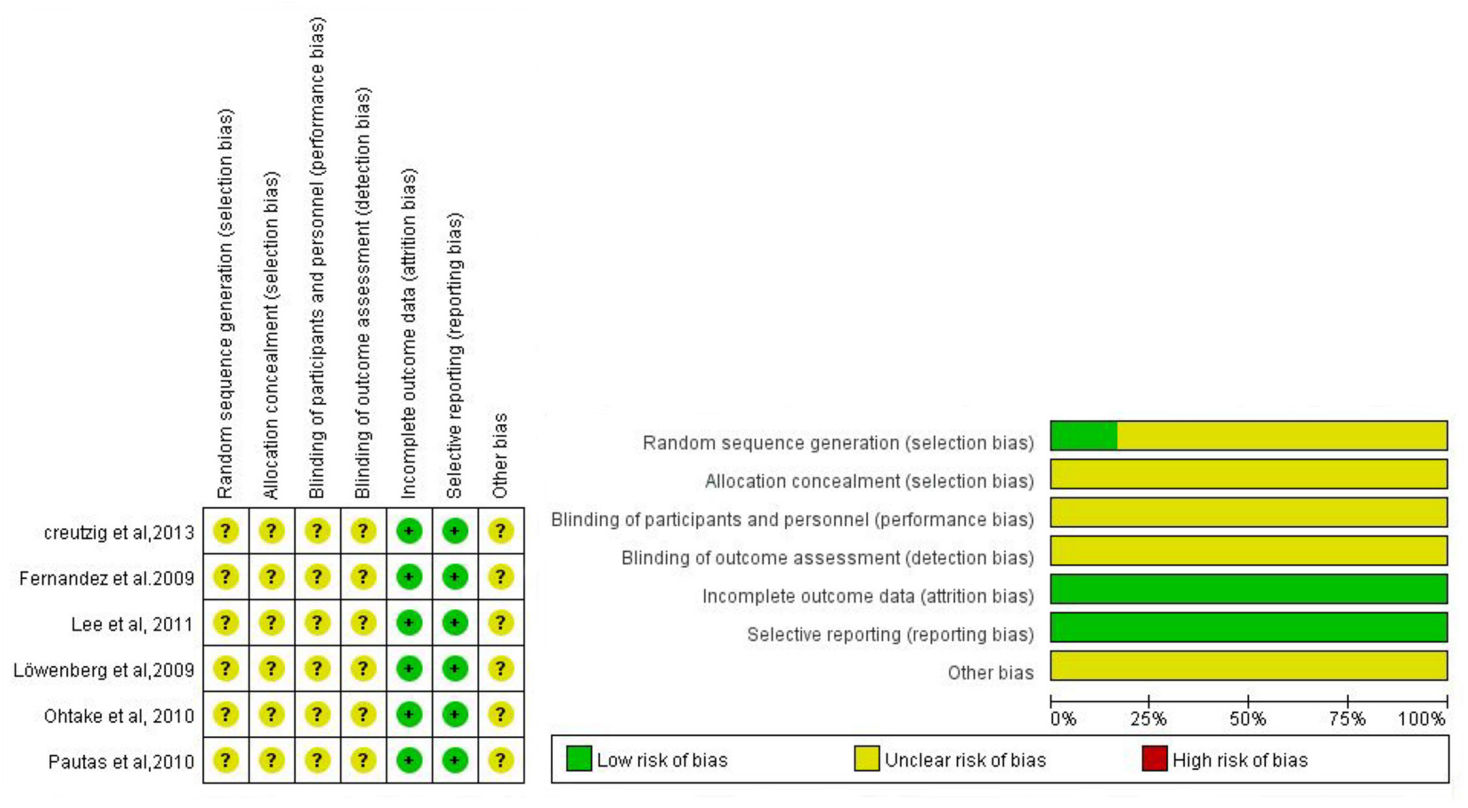
Risk of bias. This figure was the summery for risk of bias within six studies. It was formed using Revman 5.2. The definition of sources of bias and methods of assessing risk of bias can be found in Cochrane Handbook for Systematic Reviews of Interventions.

Risk of bias of the included studies is summarized in Table 2 and Fig 2. Only one study provided details on random sequence generation [10,12]. Allocation concealment and blinding of participants and personnel were not clearly reported in these studies. All studies illustrated reasons of missing data, and demonstrated that the missing data were not large enough to influence the outcome. All studies had no selected reporting.

**Table 2:** Risk of bias within studies.

Bias	Löwenberg et al, 2009	Fernandez et al, 2009	Lee et al, 2011	Creutzig et al, 2013	Ohtake et al, 2010	Pautas et al, 2010
**Random sequence generation (selection bias)**	Unclear	Unclear	Unclear	Unclear	Low risk	Unclear
**Allocation concealment (selection bias)**	Unclear	Unclear	Unclear	Unclear	Unclear	Unclear
**Blinding of participants and personnel (performance bias)**	Unclear	Unclear	Unclear	Unclear	Unclear	Unclear
**Blinding of outcome assessment (detection bias)**	Unclear	Unclear	Unclear	Unclear	Unclear	Unclear
**Incomplete outcome data (attrition bias)**	Low risk	Low risk	Low risk	Low risk	Low risk	Low risk
**Selective reporting (reporting bias)**	Low risk	Low risk	Low risk	Low risk	Low risk	Low risk
**Other bias**	Unclear	Unclear	Unclear	Unclear	Unclear	Unclear

This table was formed using Revman 5.2. The definition of sources of bias and methods of assessing risk of bias can be found in Cochrane Handbook for Systematic Reviews of Interventions.

### Results of meta-analysis

#### Complete remission

**Fig 3:**
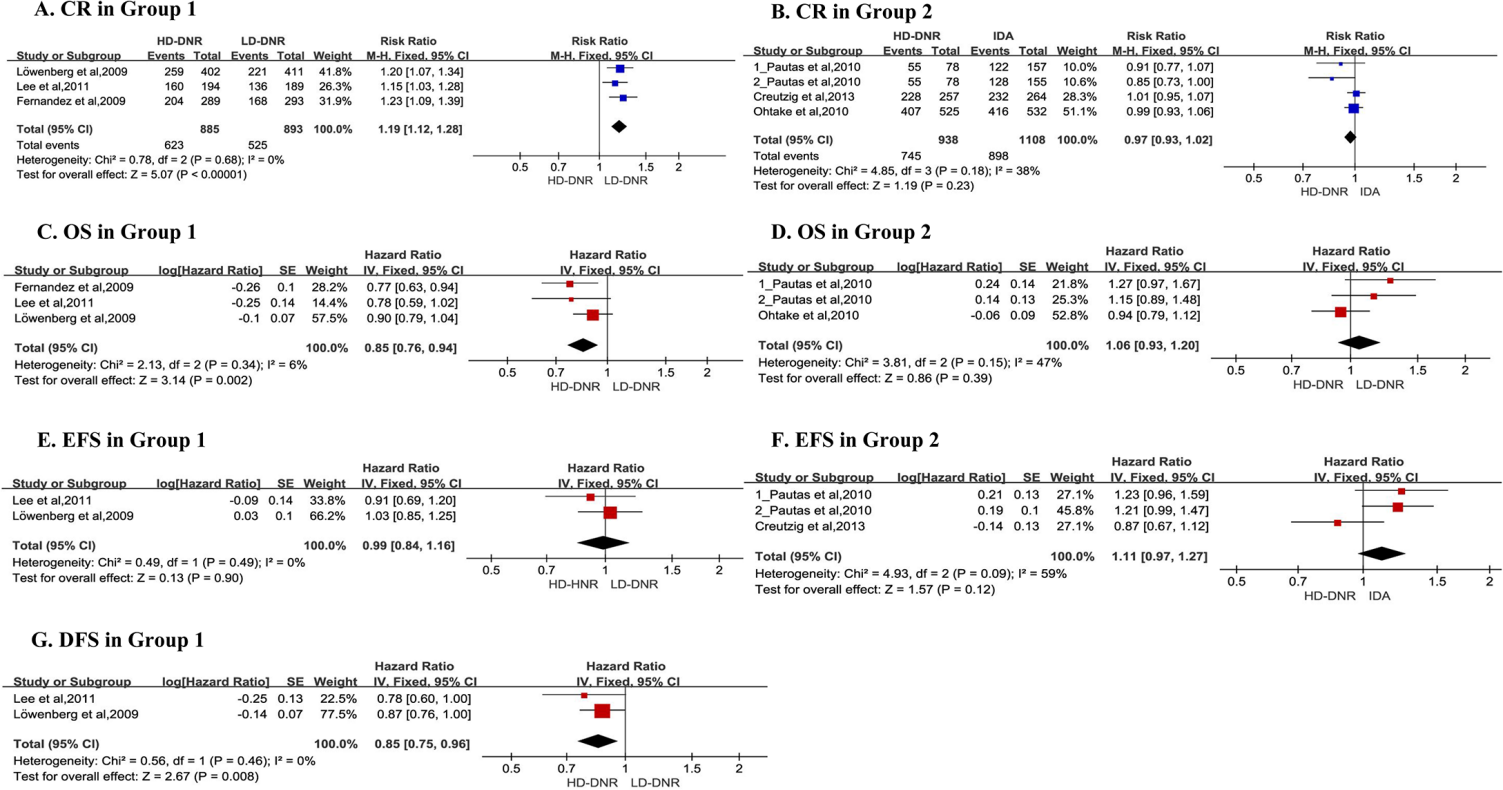
Forest plots of the RR/HR for CR, OS, EFS, DFS in Group 1 and Group 2. SE, standard error;Fixed, fixed-effect model; CI, confidence interval. Pooled RRs and HRs were computed using fixed-effect models. The size of the squares reflects each study’s relative weight, andhorizontal lines through the squares represent 95% CIs. the diamond represents the aggregate RR/HR and 95%CIs. **A. CR in Group 1** Group 1, high doses of daunorubicin vs. low doses of daunorubicin; HD-DNR, high doses of daunorubicin; LD-DNR, low doses of daunorubicin; CR, complete remission. **B. CR in Group 2** Group 2, high doses of daunorubicin vs. idarubicin; HD-DNR, high doses of daunorubicin; IDA, idarubicin; CR, complete remission. There were two subgroups named 1_Pautas et al, 2010 and2_ Pautas et al, 2010 devided from the same literature. The first one corresponded to 80×3 mg/m² of daunorubicin vs. 12×3 mg/m² of idarubicin and the second one corresponded to 80×3 mg/m² of daunorubicin vs. 12×4 mg/m² of idarubicin. **C. OS in Group 1** Group 1, high doses of daunorubicin vs. low doses of daunorubicin; HD-DNR, high doses of daunorubicin; LD-DNR, low doses of daunorubicin; OS, overall survival. **D. OS in Group 2** Group 2, high doses of daunorubicin vs. idarubicin; HD-DNR, high doses of daunorubicin; IDA, idarubicin; OS, overall survival. There were two subgroups named 1_Pautas et al, 2010 and2_ Pautas et al, 2010 devided from the same literature. The first one corresponded to 80×3 mg/m² of daunorubicin vs. 12×3 mg/m² of idarubicin and the second one corresponded to 80×3 mg/m² of daunorubicin vs. 12×4 mg/m² of idarubicin. **E. EFS in Group 1** Group 1, high doses of daunorubicin vs. low doses of daunorubicin; HD-DNR, high doses of daunorubicin; LD-DNR, low doses of daunorubicin; EFS, event-free survival. **F. EFS in Group 2** Group 2, high doses of daunorubicin vs. idarubicin; HD-DNR, high doses of daunorubicin; IDA, idarubicin; EFS, event-free survival. There were two subgroups named 1_Pautas et al, 2010 and2_ Pautas et al, 2010 devided from the same literature. The first one corresponded to 80×3 mg/m² of daunorubicin vs. 12×3 mg/m² of idarubicin and the second one corresponded to 80×3 mg/m² of daunorubicin vs. 12×4 mg/m² of idarubicin. **G. DFS in Group 1** Group 1, high doses of daunorubicin vs. low doses of daunorubicin; HD-DNR, high doses of daunorubicin; LD-DNR, low doses of daunorubicin; DFS, disease-free survival.

After induction therapy, data on complete remission were available from all three studies in Group 1[9–11]. Patients in high doses of daunorubicin arm had significantly higher complete remission than that in low doses of daunorubicin arm (RR = 1.19, 95%CI = 1.12–1.28, p<0.00001) (Fig 3A), and overall heterogeneity was not significant (I² = 0%, p = 0.68). Pooled RR for complete remission after first course of induction therapy revealed significantly higher level than that for complete remission after end of induction therapy (RR = 1.40, 95%CI = 1.28–1.54, p<0.00001), and overall heterogeneity was not significant (I² = 7%, p = 0.34). Meanwhile, the study performed subgroup analysis in accordance with age and cytogenetic risk. Pooled results suggested that complete remission from patients who are younger than 65 or with unfavourable cytogenetic risk benefited more from high doses of daunorubicin (RR = 1.24[1.15, 1.34], RR = 1.35[1.04, 1.75], respectively). And there was no significant heterogeneity.

In Group 2, data for complete remission were also extracted from all three studies [8, 12, 13]. High doses of daunorubicin did not improve complete remission (Fig 3B). Pooled RR for complete remission was 0.97 (95% CI = 0.92–1.03, p = 0.35). Heterogeneity was not significant among pooled trials (I² = 38%, p = 0.18). Similarly, complete remission after first course for patients either in high doses of daunorubicin arm or in idarubicin arm presented no significant difference (RR = 0.94[0.86, 1.01], p = 0.10) and there was no significant heterogeneity (I² = 0%, p = 0.69). According to cytogenetic risk, subgroup analysis for complete remission indicated no significant differences between high doses of daunorubicin and idarubicin with whichever kind of cytogenetic risk.

#### Overall survival

Data on overall survival were available from all three studies in Group 1 [9–11]. Patients in high doses of daunorubicin arm had significantly higher overall survival than that in low doses of daunorubicin arm (HR = 0.85, 95%CI = 0.76–0.94, p = 0.002) (Fig 3C), and overall heterogeneity was not significant (I² = 6%, p = 0.34). According to age and cytogenetic risk, the study performed subgroup analysis for overall survival. Pooled results indicated that patients younger than 65 years old or with unfavorable cytogenetic risk benefited more with high doses of daunorubicin treatment (HR = 0.76[0.65, 0.88], HR = 0.83[0.70, 0.97]).

In Group 2, data on overall survival were extracted from two studies [12, 13]. Pooled HR for overall survival was 1.06 (95%CI = 0.93–1.20, p = 0.39) (Fig 3D). Heterogeneity was not significant (I² = 47%, p = 0.15). Subgroup analysis for overall survival was not performed due to insufficient reported data.

#### Event-free survival

Data on event-free survival were available from two studies in Group 1[10, 11]. Patients in high doses of daunorubicin arm had significantly higher event-free survival than that in low doses of daunorubicin arm (HR = 0.85, 95%CI = 0.75–0.96, p = 0.008) (Fig 3E), and overall heterogeneity was not significant (I² = 0%, p = 0.46). According to cytogenetic risk, subgroup analysis manifested that patients with unfavorable cytogenetic risk benefited more in the high doses of daunorubicin arm (HR = 0.66[0.45, 0.97], p = 0.04).

In Group 2, data on event-free survival were extracted from two studies [8, 13]. Pooled HR was 1.11 (95%CI = 0.97–1.27, p = 0.12) (Fig 3F) indicating no significant difference between high doses of daunorubicin arm and idarubicin arm. Overall heterogeneity was high (I² = 59%, p = 0.09). There were not enough data in detail for advanced subgroup analysis of event-free survival.

#### Disease-free survival

Data on disease-free survival were available from two studies in Group 1[10, 11]. high doses of daunorubicin resulted in no difference in disease-free survival (Fig 3G). Pooled HR for disease-free survival was 0.99 (95%CI = 0.84–1.16, p = 0.90). Overall heterogeneity was not significant (I² = 0%, p = 0.49). No significant difference in disease-free survival was found in subgroup analysis according to cytogenetic risk.

In Group 2, data on disease-free survival were not reported in the three studies [8, 12, 13].

#### Relapse

Data on relapse were available from two studies in Group 1 [10, 11]. Patients in high doses of daunorubicin did not result in lower relapse than that in low doses of daunorubicin (Table 3). Pooled HR was 1.04 (95%CI = 0.90–1.20, p = 0.62) and overall heterogeneity was insignificant (I² = 0%, p = 0.39).

**Table 3:** Subgroup analysis for relapse and toxicity.

Meta-analysis for relapse and toxicity				
Outcome		Trials	RR M-H, Fixed, 95%CI, P value	Heterogeneity I², P value
**Group 1**	**Relapse**	2	1.04[0.90,1.20]p = 0.62	0%,p = 0.39
	**Cardiac toxic**	2	1.05[0.62,1.79]p = 0.86	0%,p = 0.46
	**Gastrointestinal toxic**	2	1.15[0.83,1.58]p = 0.40	0%,p = 0.85
	**Hemorrhage**	2	1.05[0.70,1.58]p = 0.81	0%,p = 0.32
	**Infection**	2	1.04[0.99,1.10]p = 0.13	81%,p = 0.02
**Group 2**	**Relapse**	3	0.96[0.82,1.12]p = 0.60	0%,p = 0.88
	**Cardiac toxic**	2	0.63[0.29,1.35]p = 0.23	0%,p = 0.46
	**Mucositis**	3	0.88[0.67,1.16]p = 0.37	56%,p = 0.10
	**Hemorrhage**	3	1.18[0.68,2.07]p = 0.56	0%,p = 0.69
	**Septicemia**	3	0.77[0.57,1.04]p = 0.09	42%,p = 0.18

Group 1: high doses of daunorubicin vs. low doses of daunorubicin

Group 2: high doses of daunorubicin vs. Idarubicin

RR, risk ratio; M–H, Mantel–Haenszel method; Fixed, fixed-effect model; CI, confidence interval.

In Group 2, data were extracted from two studies [8, 13]. Similar with Group 1, pooled RR resulted in insignificant difference in relapse (RR = 0.96[0.82, 1.12], p = 0.60) (Table 3).

#### Toxicity

Data on toxicity were available from two studies in Group [10, 11]. The difference was not statistically significant in cardiac toxic, gastrointestinal disease, hemorrhage, and infection (Table 3). Pooled results for toxicity in Group 2 revealed that high doses of daunorubicin was not associated with increased risk of cardiac toxic, hemorrhage, mucositis, and septicemia (Table 3).

## Discussion

Anthracyclines have been used as traditional medicine for AML induction therapy for decades. The “3+7” strategy remains the standard remission therapy. However, the right dosage of daunorubicin has been hotly debated for decades [18–23]. Based on conventional induction chemotherapy, alternative anthracyclines, intensive dose anthracycline, and additional agents like gemtuzumab ozogamicin were adopted, which were expected to achieve higher complete remission and survival rate [18–20, 22].

A large number of clinical trials of strengthening doses of DNR were conducted to compare with traditional doses of daunorubicin or other anthracycline (such as idarubicin) at equivalent doses [18–20, 22]. The trials which used a high dose of daunorubicin 70–95 mg/m² for 3 days conducted by the Southwest Oncology Group, the Acute Leukemia French Association (ALFA), and CALGB, showed high rates of complete remission with acceptable toxicity [24–26]. Results from a large number of randomized controlled trials conducted by the Eastern Cooperative Oncology Group (ECOG) indicated that to intensify the dose of daunorubicin from 45 to 90 mg/m²for 3 days could achieve higher rates of complete remission (70.6% vs. 57.3%, p<0.001) and improved overall survival (median, 23.7 vs. 15.7 months, p = 0.003) for AML patients who were younger than 60 (Fernandez and Sun et al., 2009). Another large randomized controlled trials which focused on old patients with AML demonstrated that patients aged 60–65 in high-dose daunorubicin group (90 mg/m²), as compared with those in the same age group who received traditional-dose daunorubicin (45 mg/m²), had higher rates of complete remission (73% vs. 51%), overall survival (38% vs. 23%) and event-free survival (29% vs. 14%) [11]. A meta-analysis showed that high doses of daunorubicin, compared to low doses of daunorubicin, was associated with reduced rates of remission failure and overall mortality [27]. However, comparing with standard-dose of idarubicin (cumulative dose, 36–48 mg/m²), some other trials failed to show the benefit from high-dose of daunorubicin (cumulative dose, 240–250 mg/m²) [8, 12, 13]. A meta-analysis showed better overall survival on adult patients who received idarubicin than those receiving at least 180mg/m^2^ of daunorubicin [1].

In order to resolve the above conflict evidence in induction treatment of AML, a systematic review and meta-analysis was conducted. Our meta-analysis results suggested that comparing with low doses of daunorubicin, high doses of daunorubicin improved about 15% overall survival (HR = 0.85, 95%CI = 0.76–0.94), 15% event-free survival(HR = 0.85, 95%CI = 0.75–0.96), and 19% complete remission (RR = 1.19, 95%CI = 1.12–1.28). RR for complete remission after first course was even higher (RR = 1.40, 95%CI = 1.28–1.54). However, there is no significant difference in disease-free survival (HR = 0.99,95%CI = 0.84–1.16) and relapse (RR = 1.04,95%CI = 0.90–1.20). These results raised a controversial conclusion that high doses of daunorubicin may improve overall survival and event-free survival, but increase the incidence of risk ratio. This may be due to the inclusion of different studies with different outcomes in the meta-analysis.

In addition, subgroup analyses were performed for major outcomes. Though AML-related prognostic factors include white blood cell (WBC) count,age, cytogenetic factors, molecular factors, performance status, existence of prior MDS, this study only considered age and cytogenetic factors because data for other prognostic factors were insufficient (Tables 4 and 5). According to age, complete remission and overall survival of those younger than 65 years old were improved (RR = 1.24[1.15, 1.34], p<0.00001; HR = 0.76[0.65, 0.88], p = 0.0003) by using high doses of daunorubicin, but no clinical benefits were documented in older patients (>65yr). According to cytogenetic risk, high doses of daunorubicin increased complete remission in all three cytogenetic subgroups (favourable, intermediate, and unfavourable risk groups) especially in the unfavourable risk group (RR = 1.35[1.04, 1.75], p = 0.03). HR for overall survival and event-free survival were significant only in the unfavourable risk group (HR = 0.83[0.70, 0.97], p = 0.02; HR = 0.66[0.45, 0.97], p = 0.04). Generally speaking, the above results indicate that high doses of daunorubicin accelerates overall complete remission, overall survival and event-free survival. Patients younger than 65yrs or with unfavorable cytogenetic risk are suggested to choose high doses of daunorubicin rather than low doses of daunorubicin.

**Table 4:** Subgroup analysis for CR (complete remission).

Subgroup Analysis for CR				
Outcome	Subgroup	Trials	RR M-H, Fixed, 95%CI, P value	Heterogeneity I², P value
**CR according to age in Group 1**	<65 years old	3	1.24[1.15,1.34]p<0.00001	55%,p = 0.11
	>65 years old	1	1.08[0.93,1.25]p = 0.34	not applicable
**CR according to cytogenetic risk in Group 1**	favorable risk	2	1.14[1.01,1.29]p = 0.03	0%,p = 0.39
	intermediate risk	2	1.11[1.00,1.23]p = 0.04	0%,p = 0.87
	unfavorable risk	2	1.35[1.04,1.75]p = 0.02	0%,p = 0.60
**CR according to cytogenetic risk in Group 2**	favorable risk	3	1.06[0.99,1.13]p = 0.09	0%,p = 0.88
	intermediate risk	3	0.94[0.88,1.01]p = 0.11	0%,p = 0.72
	unfavorable risk	3	0.80[0.59,1.08]p = 0.14	0%,p = 0.81

Group 1, high doses of daunorubicin vs. low doses of daunorubicin

Group 2, high doses of daunorubicin vs. Idarubicin

RR, risk ratio; M–H, Mantel–Haenszel method; Fixed, fixed-effect model; CI, confidence interval.

**Table 5:** Subgroup analysis for survival data.

Subgroup analysis for survival data				
Outcome	Subgroup	Trials	HR M-H, Fixed, 95%CI, P value	Heterogeneity I², P value
**OS according to age in Group 1**	15–65 years old	3	0.76[0.65,0.88]p = 0.0003	0%,p = 0.79
	65–83 years old	1	1.09[0.92,1.31]p = 0.32	not applicable
**OS according to cytogenetic risk in Group 1**	favorable risk	2	0.85[0.44,1.64]p = 0.62	68%,p = 0.08
	intermediate risk	2	0.87[0.71,1.06]p = 0.16	57%,p = 0.13
	unfavorable risk	3	0.83[0.70,0.97]p = 0.02	48%,p = 0.15
**EFS according to cytogenetic risk in Group 1**	favorable risk	2	0.85[0.47,1.53]p = 0.59	63%,p = 0.10
	intermediate risk	2	0.90[0.76,1.07]p = 0.24	62%,p = 0.10
	unfavorable risk	2	0.66[0.45,0.97]p = 0.04	0%,p = 0.35
**DFS according to cytogenetic risk in Group 1**	favorable risk	2	1.52[1.00,2.30]p = 0.05	75%,p = 0.04
	intermediate risk	2	0.93[0.76,1.15]p = 0.53	67%,p = 0.08
	unfavorable risk	2	0.84[0.51,1.38]p = 0.49	48%,p = 0.16

OS, overall survival; DFS, disease-free survival; EFS, event-free survival

Group 1, high doses of daunorubicin vs. low doses of daunorubicin

Group 2, high doses of daunorubicin vs. Idarubicin

HR, hazard ratio; M–H, Mantel–Haenszel method; Fixed, fixed-effect model; CI, confidence interval.

Compared with idarubicin, there were no significant differences in primary and secondary outcomes. Therefore, considering either efficacy or safety, high doses of daunorubicin and idarubicin can be chosen as an induction therapy for AML.

In high doses of daunorubicin group of all six included studies, cumulative dosage of daunorubicin were from 240 to 270 mg/m² with Ara-C from 100 to 200×7 mg/m²×days during induction therapy. Induction drug administration of controlled groups was also similar to each other in Group 1 and Group 2. In Pautas et al. (2010), three patients’ groups were treated by 80×3 mg/m² of daunorubicin, and 12×3 or 12×4 mg/m² of idarubicin. The trial was divided into two parts, namely 80×3 mg/m² of daunorubicin vs. 12×3 mg/m² of idarubicin, and 80×3 mg/m² of daunorubicin, 12×4 mg/m² of idarubicin. Meanwhile, subjects using 80×3 mg/m² of daunorubicin in the primary group were divided by half forming two new trials to guarantee consistency of the total sample.

In subgroup analysis, for Group 1, based on cytogenetic risk, heterogeneity of overall survival and disease-free survival appeared high when patients were classified into subgroups. The main reason for the high heterogeneity is most likely patients’ age. Lee et al mainly studied adults aged 17–60 yrs whileas older patients aged 60–83 were included in Löwenberg et al’s trial. The patients’ response to medicine and tolerance to side reaction differed in different age groups. According to data in forest plots, for old patients with favorable risk, high dose of daunorubicin could prolong overall survival and disease-free survival compared with traditional dose of daunorubicin. But for old patients with intermediate or unfavorable risk, superiority of high doses of daunorubicin for survival was not shown, which might be caused by severer side effects [11]. On the contrary, for young patients with favorable risk, traditional dose of daunorubicin achieved better overall survival and disease-free survival on the contrary. For young patients with intermediate risk and unfavorable risk, this condition was opposite. Due to the small number of studies included, meta-analysis could not provide stratified therapeutic schedule according to cytogenetic risk in detail.

The safety issue of high doses of daunorubicin always needs to be considered. Cardiac toxic, gastrointestinal toxic, hemorrhage and infection are common toxicity of anthracycline treatment. Usually it was considered that with the dose escalation of drugs, the rate of toxicity will be increased, too. However, we are very pleased to see that the rate of cardiac toxic, gastrointestinal toxic, hemorrhage and infection in the two comparisons, were similar, which indicated that high doses of daunorubicin is as safe as other therapy.

Something must be pointed out that to increase single and cumulative dosage of daunorubicin without increasing cardiotoxicity for pediatric AML, Creutzig et al adopted liposomal formulation of daunorubicin while others reported not. The differences both in this special medicine formulation and in younger age may lead to bias when pooling the data,such as the heterogeneity(I = 59%, p = 0.09) shown in Fig 3F. So we removed this trial from meta-analysis to reflect the influence of the individual data on the pooled RRs/HRs, and the corresponding pooled results were not materially altered (data not shown).

There are some limitations of this meta-analysis. Firstly, though these randomized controlled trials are of good quality with large sample sizes and published in authentic journals, only six trials were included in this meta-analysis. Secondly, more detailed data analyses of patients’ age, cytogenetic risk and other prognostic factors (such as performance status, FAB classification and so on) should be used to update the results and provide the best choice for individualized treatment. For example, data from Eastern Cooperative Oncology Trial E1900 was updated recently (American Society of Hematology, 2014, abstracts) and benefit of high doses of daunorubicin in AML patients with FLT3-TD, NPM1, and DNMT3A mutant was confirmed after a long time follow-up. We hope there will be more related large randomized controlled trials to conduct further meta-anlysis. Thirdly, data on induction death or early death could not be pooled as the definitions were different in included studies. Last but not the least, like most of the published meta-analyses, our analysis was based on summary data rather than individual patient data. Thus, it could not produce merged survival curves and explore patient-level factors which may be responsible for the variations of treatment effects.

In conclusion, the analysis indicated that compared with low doses of daunorubicin, high doses of daunorubicin improves complete remission, disease-free survival and event-free survival without increasing toxicity rates for newly diagnosed AML, especially for young patients (< 65yrs) or patients with unfavorable cytogenetic risk. However, there is no difference between high dose of daunorubicin and idarubicin. These results were in accordance with NCCN 2013 which formulated the strategy based on a single RCT. This study needs further high quality randomized controlled trials to strengthen the evidence.

## Supporting Information

S1 Table PRISMA 2009 Checklist. (PDF)

S1 Text Search strategy. (PDF)

## References

[1] Wang J, Yang YG, Zhou M, Xu JY, Zhang QG, Zhou RF, et al. Meta-analysis of randomised clinical trials comparing idarubicin + cytarabine with daunorubicin + cytarabine as the induction chemotherapy in patients with newly diagnosed acute myeloid leukaemia. 2013. p. e60699. 10.1371/journal.pone.0060699 PMC3622517 23593285

[2] Roboz GJ. Novel approaches to the treatment of acute myeloid leukemia. Hematology Am Soc Hematol Educ Program 2011;2011:43–50. 10.1182/asheducation-2011.1.43 22160011

[3] Dohner H, Estey EH, Amadori S, Appelbaum FR, Buchner T, Burnett AK, et al Diagnosis and management of acute myeloid leukemia in adults: recommendations from an international expert panel, on behalf of the European LeukemiaNet. Blood 2010;115:453–74. 10.1182/blood-2009-07-235358 19880497

[4] Estey E, Dohner H. Acute myeloid leukaemia. Lancet 2006;368:1894–907. 17126723 10.1016/S0140-6736(06)69780-8

[5] Lowenberg B, Griffin JD, Tallman MS. Acute myeloid leukemia and acute promyelocytic leukemia. Hematology Am Soc Hematol Educ Program 2003:82–101. 14633778

[6] Buchner T, Berdel WE, Haferlach C, Haferlach T, Schnittger S, Muller-Tidow C, et al Age-related risk profile and chemotherapy dose response in acute myeloid leukemia: a study by the German Acute Myeloid Leukemia Cooperative Group. J Clin Oncol 2009;27:61–9. 10.1200/JCO.2007.15.4245 19047294

[7] Burnett AK, Hills RK, Milligan DW, Goldstone AH, Prentice AG, McMullin MF, et al Attempts to optimize induction and consolidation treatment in acute myeloid leukemia: results of the MRC AML12 trial. J Clin Oncol 2010;28:586–95. 10.1200/JCO.2009.22.9088 20038732

[8] Creutzig U, Zimmermann M, Bourquin JP, Dworzak MN, Fleischhack G, Graf N, et al Randomized trial comparing liposomal daunorubicin with idarubicin as induction for pediatric acute myeloid leukemia: results from Study AML-BFM 2004. Blood 2013;122:37–43. 10.1182/blood-2013-02-484097 23704089

[9] Fernandez HF, Sun Z, Yao X, Litzow MR, Luger SM, Paietta EM, et al Anthracycline Dose Intensification in Acute Myeloid Leukemia. The New England Journal of Medicine 2009;361:1249–59. 10.1056/NEJMoa0904544 19776406 PMC4480917

[10] Lee JH, Joo YD, Kim H, Bae SH, Kim MK, Zang DY, et al A randomized trial comparing standard versus high-dose daunorubicin induction in patients with acute myeloid leukemia. Blood 2011;118:3832–41. 10.1182/blood-2011-06-361410 21828126

[11] Lowenberg B, Ossenkoppele GJ, van Putten W, Schouten HC, Graux C, Ferrant A, et al High-dose daunorubicin in older patients with acute myeloid leukemia. N Engl J Med 2009;361:1235–48. 10.1056/NEJMoa0901409 19776405

[12] Ohtake S, Miyawaki S, Fujita H, Kiyoi H, Shinagawa K, Usui N, et al Randomized study of induction therapy comparing standard-dose idarubicin with high-dose daunorubicin in adult patients with previously untreated acute myeloid leukemia: the JALSG AML201 Study. Blood 2011;117:2358–65. 10.1182/blood-2010-03-273243 20693429

[13] Pautas C, Merabet F, Thomas X, Raffoux E, Gardin C, Corm S, et al Randomized study of intensified anthracycline doses for induction and recombinant interleukin-2 for maintenance in patients with acute myeloid leukemia age 50 to 70 years: results of the ALFA-9801 study. J Clin Oncol 2010;28:808–14. 10.1200/JCO.2009.23.2652 20048183

[14] Moher D, Liberati A, Tetzlaff J, Altman DG. Preferred reporting items for systematic reviews and meta-analyses: the PRISMA statement. Int J Surg 2010;8:336–41. 10.1016/j.ijsu.2010.02.007 20171303

[15] Cheson BD, Bennett JM, Kopecky KJ, Buchner T, Willman CL, Estey EH, et al Revised recommendations of the International Working Group for Diagnosis, Standardization of Response Criteria, Treatment Outcomes, and Reporting Standards for Therapeutic Trials in Acute Myeloid Leukemia. J Clin Oncol 2003;21:4642–9. 14673054 10.1200/JCO.2003.04.036

[16] Tierney JF, Stewart LA, Ghersi D, Burdett S, Sydes MR. Practical methods for incorporating summary time-to-event data into meta-analysis. Trials 2007;8:16 17555582 10.1186/1745-6215-8-16 PMC1920534

[17] Higgins JP, Thompson SG. Quantifying heterogeneity in a meta-analysis. Stat Med 2002;21:1539–58. 12111919 10.1002/sim.1186

[18] Berman E, Heller G, Santorsa J, McKenzie S, Gee T, Kempin S, et al Results of a randomized trial comparing idarubicin and cytosine arabinoside with daunorubicin and cytosine arabinoside in adult patients with newly diagnosed acute myelogenous leukemia. Blood 1991;77:1666–74. 2015395

[19] Mandelli F, Petti MC, Ardia A, Di Pietro N, Di Raimondo F, Ganzina F, et al A randomised clinical trial comparing idarubicin and cytarabine to daunorubicin and cytarabine in the treatment of acute non-lymphoid leukaemia. A multicentric study from the Italian Co-operative Group GIMEMA. Eur J Cancer 1991;27:750–5. 1829918 10.1016/0277-5379(91)90181-c

[20] Vogler WR, Velez-Garcia E, Weiner RS, Flaum MA, Bartolucci AA, Omura GA, et al A phase III trial comparing idarubicin and daunorubicin in combination with cytarabine in acute myelogenous leukemia: a Southeastern Cancer Study Group Study. J Clin Oncol 1992;10:1103–11. 1607916 10.1200/JCO.1992.10.7.1103

[21] Wheatley K. A systematic collaborative overview of randomized trials comparing idarubicin with daunorubicin (or other anthracyclines) as induction therapy for acute myeloid leukaemia. AML Collaborative Group. Br J Haematol 1998;103:100–9. 9792296

[22] Wiernik PH, Banks PL, Case DJ, Arlin ZA, Periman PO, Todd MB, et al Cytarabine plus idarubicin or daunorubicin as induction and consolidation therapy for previously untreated adult patients with acute myeloid leukemia. Blood 1992;79:313–9. 1730080

[23] Ziogas DC, Voulgarelis M, Zintzaras E. A network meta-analysis of randomized controlled trials of induction treatments in acute myeloid leukemia in the elderly. Clin Ther 2011;33:254–79. 10.1016/j.clinthera.2011.04.004 21600383

[24] Appelbaum FR, Dahlberg S, Thomas ED, Buckner CD, Cheever MA, Clift RA, et al. Bone marrow transplantation or chemotherapy after remission induction for adults with acute nonlymphoblastic leukemia. A prospective comparison Results of a randomized trial comparing idarubicin and cytosine arabinoside with daunorubicin and cytosine arabinoside in adult patients with newly diagnosed acute myelogenous leukemia. 1984. p. 581–8. 10.7326/0003-4819-101-5-581 6385797

[25] Castaigne S, Chevret S, Archimbaud E, Fenaux P, Bordessoule D, Tilly H, et al Randomized comparison of double induction and timed-sequential induction to a "3 + 7" induction in adults with AML: long-term analysis of the Acute Leukemia French Association (ALFA) 9000 study. Blood 2004;104:2467–74. 15142880 10.1182/blood-2003-10-3561

[26] Kolitz JE, George SL, Dodge RK, Hurd DD, Powell BL, Allen SL, et al Dose escalation studies of cytarabine, daunorubicin, and etoposide with and without multidrug resistance modulation with PSC-833 in untreated adults with acute myeloid leukemia younger than 60 years: final induction results of Cancer and Leukemia Group B Study 9621. J Clin Oncol 2004;22:4290–301. 15514371 10.1200/JCO.2004.11.106

[27] Teuffel O, Leibundgut K, Lehrnbecher T, Alonzo TA, Beyene J, Sung L. Anthracyclines during induction therapy in acute myeloid leukaemia: a systematic review and meta-analysis. British Journal of Haematology 2013;161:192–203. 10.1111/bjh.12233 23398482

