# Supplementary material for: High Doses of Daunorubicin during Induction Therapy of Newly Diagnosed Acute Myeloid Leukemia: A Systematic Review and Meta-Analysis of Prospective Clinical Trials
Source: PLoS One. 2015 May 20;10(5):e0125612. doi: 10.1371/journal.pone.0125612 (PMC4439155; doi:10.1371/journal.pone.0125612)
Supplement: S1 Text — (PDF) [file pone.0125612.s002.pdf]

appendix 1

Cochrane library search strategy

1、MeSH descriptor: [Leukemia, Myeloid, Acute] explode all trees
2、MeSH descriptor: [Leukemia, Myeloid] explode all trees
3、MeSH descriptor: [Acute Disease] explode all trees
4、#2 and #3
5、(acut* or akut* or agud* or aigu*):ti,ab,kw
6、myelo* or mielo* or nonlympho* or granulocytic*) and (leuk*em* or leuc*)):ti,ab,kw
7、#5 and #6
8、aml*:ti,ab,kw
9、anll:ti,ab,kw
10、#1 or #4 or #7 or #8 or #9
11、MeSH descriptor: [Daunorubicin] explode all trees
12、Daunomycin:ti,ab,kw
13、Rubomycin:ti,ab,kw
14、Rubidomycin:ti,ab,kw
15、Dauno-Rubidomycine:ti,ab,kw
16、NSC-82151:ti,ab,kw
17、NSC82151:ti,ab,kw
18、Daunoblastin:ti,ab,kw
19、Daunoblastine:ti,ab,kw
20、Cerubidine:ti,ab,kw
21、Daunorubicin Hydrochloride:ti,ab,kw
22、Hydrochloride, Daunorubicin:ti,ab,kw
23、Daunorubicin:ti,ab,kw
24、#12 or #13 or #14 or #15 or #16 or #17 or #18 or #19 or #20 or #21 or #22 or #23
25、#11 or #24
26、#10 and #25
27、random*:ti,ab,kw
28、Placebo*:ti,ab,kw
29、'single':ab,ti or 'double':ab,ti or 'triple':ab,ti or 'treble':ab,ti
30、'blinded':ab,ti or 'masked':ab,ti
31、#29 and #30
32、'Controlled clinical trial':ab,ti
33、#27 or #28 or #31 or #32
34、#26 and #33

Pubmed search strategy

#22 Search ((#21) AND #10) AND #9
#21 Search (#19) AND #20
#20 Search humans[mh]
#19 Search ((((((#11) OR #12) OR #13) OR #14) OR #15) OR #16) OR #17) OR #18
#18 Search groups[tiab]
#17 Search trial[tiab]
#16 Search randomly[tiab]
#15 Search drug therapy[sh]
#14 Search placebo[tiab]
#13 Search randomized[tiab]
#12 Search controlled clinical trial[pt]
#11 Search randomized controlled trial[pt]
#10 Search (((((((((((((Daunomycin[Title/Abstract]) OR Rubomycin[Title/Abstract]) OR Rubidomycin[Title/Abstract]) OR Dauno-Rubidomycine[Title/Abstract]) OR Dauno Rubidomycine[Title/Abstract]) OR NSC-82151[Title/Abstract]) OR NSC 82151[Title/Abstract]) OR NSC82151[Title/Abstract]) OR Daunoblastin[Title/Abstract]) OR Daunoblastine[Title/Abstract]) OR Cerubidine[Title/Abstract]) OR Daunorubicin Hydrochloride[Title/Abstract]) OR Hydrochloride
#9 Search ((#8) OR #4) OR #1
#8 Search ((#7) AND #6) AND #5
#7 Search (leuk*em*[Title/Abstract]) OR leuc*[Title/Abstract]
#6 Search (((myelo*[Title/Abstract]) OR mielo*[Title/Abstract]) OR nonlympho[Title/Abstract]) OR granulocytic*
#5 Search (((acut*[Title/Abstract]) OR akut*[Title/Abstract]) OR agud*[Title/Abstract]) OR aigu*[Title/Abstract]
#4 Search (#2) AND #3
#3 Search acute disease[MeSH Terms]
#2 Search leukemia
#1 Search Leukemia

Embase search strategy

#35. acute AND granulocytic AND leukemia:exp OR (myeloid AND leukemia:de AND acute AND disease:de) OR (acut*:ab,ti OR akut*:ab,ti OR agud*:ab,ti OR aigu*:ab,ti AND (myelo*:ab,ti OR mielo*:ab,ti OR nonlympho*:ab,ti OR granulocytic*:ab,ti) AND (leuk*em*:ab,ti OR leuc*:ab,ti)) OR aml:ab,ti OR anll:ab,ti AND (daunomycin:ab,ti OR rubomycin:ab,ti OR rubidomycin:ab,ti OR 'dauno rubidomycine':ab,ti OR 'nsc 82151':ab,ti OR nsc82151:ab,ti OR daunoblastin:ab,ti OR daunoblastine:ab,ti OR cerubidine:ab,ti OR 'daunorubicin hydrochloride':ab,ti OR 'hydrochloride daunorubicin':ab,ti OR daunorubicin:ab,ti) AND (random*:ab,ti OR placebo*:ab,ti OR ('single':ab,ti OR 'double':ab,ti OR 'triple':ab,ti OR 'treble':ab,ti AND ('blinded':ab,ti OR 'masked':ab,ti)) OR 'controlled clinical trial':ab,ti OR 'retracted article':de) NOT ('animal':de NOT 'human':de)
#34. random*:ab,ti OR placebo*:ab,ti OR ('single':ab,ti OR 'double':ab,ti OR 'triple':ab,ti OR 'treble':ab,ti AND ('blinded':ab,ti OR 'masked':ab,ti)) OR 'controlled clinical trial':ab,ti OR 'retracted article':de NOT ('animal':de NOT 'human':de)

#33. 'animal':de NOT 'human':de
#32. random*:ab,ti OR placebo*:ab,ti OR ('single':ab,ti OR 'double':ab,ti OR 'triple':ab,ti OR 'treble':ab,ti AND ('blinded':ab,ti OR 'masked':ab,ti)) OR 'controlled clinical trial':ab,ti OR 'retracted article':de
#31. 'retracted article':de
#30. 'controlled clinical trial':ab,ti
#29. 'single':ab,ti OR 'double':ab,ti OR'triple':ab,ti OR 'treble':ab,ti AND ('blinded':ab,ti OR 'masked':ab,ti)
#28. 'blinded':ab,ti OR 'masked':ab,ti
#27. 'single':ab,ti OR 'double':ab,ti OR 'triple':ab,ti OR 'treble':ab,ti
#26. placebo*:ab,ti
#25. random*:ab,ti
#24. acute AND granulocytic AND leukemia:exp OR (myeloid AND leukemia:de AND acute AND disease:de) OR (acut*:ab,ti OR akut*:ab,ti OR agud*:ab,ti OR aigu*:ab,ti AND (myelo*:ab,ti OR mielo*:ab,ti OR nonlympho*:ab,ti OR granulocytic*:ab,ti) AND (leuk*em*:ab,ti OR leuc*:ab,ti)) OR aml:ab,ti OR anll:ab,ti AND (daunomycin:ab,ti OR rubomycin:ab,ti OR rubidomycin:ab,ti OR 'dauno rubidomycine':ab,ti OR 'nsc 82151':ab,ti OR nsc82151:ab,ti OR daunoblastin:ab,ti OR daunoblastine:ab,ti OR cerubidine:ab,ti OR 'daunorubicin hydrochloride':ab,ti OR 'hydrochloride daunorubicin':ab,ti OR daunorubicin:ab,ti)
#23. daunomycin:ab,ti OR rubomycin:ab,ti OR rubidomycin:ab,ti OR 'dauno rubidomycine':ab,ti OR 'nsc 82151':ab,ti OR nsc82151:ab,ti OR daunoblastin:ab,ti OR daunoblastine:ab,ti OR cerubidine:ab,ti OR 'daunorubicin hydrochloride':ab,ti OR 'hydrochloride daunorubicin':ab,ti OR daunorubicin:ab,ti
#22. daunorubicin:ab,ti
#21. 'hydrochloride daunorubicin':ab,ti
#20. 'daunorubicin hydrochloride':ab,ti
#19. cerubidine:ab,ti
#18. daunoblastine:ab,ti
#17. daunoblastin:ab,ti
#16. nsc82151:ab,ti
#15. 'nsc 82151':ab,ti
#14. 'dauno rubidomycine':ab,ti
#13. rubidomycin:ab,ti
#12. rubomycin:ab,ti
#11. daunomycin:ab,ti
#10. acute AND granulocytic AND leukemia:exp OR (myeloid AND leukemia:de AND acute AND disease:de) OR (acut*:ab,ti OR akut*:ab,ti OR agud*:ab,ti OR aigu*:ab,ti AND (myelo*:ab,ti OR mielo*:ab,ti OR nonlympho*:ab,ti OR granulocytic*:ab,ti) AND (leuk*em*:ab,ti OR leuc*:ab,ti)) OR aml:ab,ti OR anll:ab,ti
#9. anll:ab,ti
#8. aml:ab,ti
#7. acut*:ab,ti OR akut*:ab,ti OR agud*:ab,ti OR aigu*:ab,ti AND (myelo*:ab,ti OR mielo*:ab,ti OR nonlympho*:ab,ti OR granulocytic*:ab,ti) AND (leuk*em*:ab,ti OR leuc*:ab,ti)
#6. myelo*:ab,ti OR mielo*:ab,ti OR nonlympho*:ab,ti OR granulocytic*:ab,ti AND

(leuk*em*:ab,ti OR leuc*:ab,ti)

#5. acut*:ab,ti OR akut*:ab,ti OR agud*:ab,ti OR aigu*:ab,ti

#4. myeloid AND leukemia:de AND acute AND disease:de

#3. acute AND disease:de

#2. myeloid AND leukemia:de

#1. acute AND granulocytic AND leukemia:exp
